# Awareness, Knowledge, and Attitudes of Dentists in Türkiye Regarding the 2034 Phase-Out of Dental Amalgam Under the Minamata Convention: A Cross-Sectional Study

**DOI:** 10.3290/j.ohpd.c_2804

**Published:** 2026-09-09

**Authors:** Ayşenur Altuğ Yıldırım, Sena Ayyıldız

**Affiliations:** a Ayşenur Altuğ Yıldırım Assistant Professor, Department of Restorative Dentistry , Faculty of Dentistry , Çankırı Karatekin University, Çankırı, Türkiye. Conceptualization, methodology, investigation, data collection, formal analysis, visualization, wrote original draft, edited the manuscript, reviewed and approved the final version of the manuscript; b Sena Ayyıldız Assistant Professor, Department of Pediatric Dentistry, Faculty of Dentistry, Çankırı Karatekin University, Çankırı, Türkiye. Methodology, investigation, data collection, edited the manuscript, reviewed and approved the final version of the manuscript.

**Keywords:** dental amalgam, Minamata Convention, phase-down.

## Abstract

**Purpose:**

This study evaluated Turkish dentists’ knowledge of and attitudes toward the Minamata Convention, the 2025 amalgam phase-down decisions, and current dental amalgam use, and identified factors associated with knowledge and attitude scores across sociodemographic and professional subgroups.

**Materials and Methods:**

A cross-sectional survey was conducted among 408 dentists practising in Türkiye between January and April 2026. The 26-item questionnaire assessed sociodemographic and professional characteristics, amalgam use frequency, perceived health and environmental risks, awareness of the Minamata Convention and the 2025 regulatory amendments, factual knowledge, and attitudes toward amalgam phase-down. Internal consistency was evaluated using Cronbach’s alpha. Group comparisons were performed using independent-samples t-tests, Welch’s ANOVA with Games–Howell post-hoc tests, and Pearson’s correlation. Effect sizes were reported as Cohen’s d and eta-squared.

**Results:**

Only 40.2% had heard of the Minamata Convention, and 21.1% were aware of the 2025 phase-down decisions. Knowledge and attitude scores were positively correlated (r = 0.242, p < 0.001). Knowledge scores were significantly higher among academic staff, university-based practitioners, and those aware of the Convention (all p < 0.001). Attitude scores were significantly higher among females (d = 0.61), non-users of amalgam, and those with Minamata awareness. Most participants (73.0%) never used amalgam, yet 79.9% perceived it as an environmental risk.

**Conclusions:**

Despite favorable attitudes toward amalgam reduction, knowledge of the Minamata Convention remains limited among Turkish dentists. Targeted educational programs are needed, particularly for general practitioners in private practice, to support the transition toward mercury-free restorative dentistry.

For over a century, dental amalgam has served as one of the most widely used direct restorative materials in dentistry, valued for its mechanical strength, longevity, ease of manipulation, and favorable performance in stress-bearing posterior cavities.^7,14,21^ Resin-based composites and other tooth-colored restorative materials are available; however, dental amalgam continues to be used frequently in certain situations: patients at high risk for caries, inadequate moisture control situations, or limited access to dental care settings.^12,22^ However, the mercury content of dental amalgams has also raised concerns egarding its effects on environmental and public health.^2,5,6,15,23^

Mercury is a globally recognized environmental pollutant with well-documented toxic effects on human health and ecosystems.^4,19,30^ In response to increasing awareness of mercury-related risks and recognising the urgency of addressing mercury exposure at a global level, the international community negotiated and adopted the Minamata Convention on Mercury in 2013, establishing a legally binding framework to curtail anthropogenic mercury emissions and safeguard both public health and ecological systems.^27^ Convention takes its name from the Minamata disease outbreak in Japan, which highlighted the devastating consequences of mercury contamination.^16^ The dental amalgam has been explicitly included under the convention as a controlled product containing mercury. Since its entry into force in 2017, the Convention has been ratified by more than 150 Parties worldwide, including Türkiye (signed in September 2014 and ratified it in October 2022) demonstrating a broad international commitment to reducing mercury-related risks.^26^

Rather than enforcing an immediate prohibition, the Convention’s initial approach entailed a graduated reduction or “phase-down” of dental amalgam use, emphasizing gradual reduction through preventive dentistry, promotion of alternative restorative materials, restrictions on use in vulnerable populations, and the implementation of environmentally responsible waste management practices such as dental amalgam separators.^18^ This was significantly pushed forward during the Sixth Meeting of the Conference of the Parties (COP-6), which took place in Geneva in November 2025.29 As part of COP 6, amendments establishing a time-bound global phase-out of dental amalgam by 2034 were adopted, marking a historic turning point in Minamata Convention implementation.^28,29^

The 2025 decisions reiterated prioritisation of mercury-free materials in routine clinical practice, the reinforcement of population-level preventive strategies, and the adoption of targeted protective measures for groups considered particularly susceptible to mercury exposure notably pregnant and lactating women and paediatric patients. In parallel, the parties strengthened obligations concerning the environmentally sound management of dental amalgam, including restricting its use to encapsulated forms.^11,28,29^

These developments position dentists and dental education institutions as the primary executors of the Minamata Convention. Dentists are involved not only in clinical choices about restorative materials but also in areas concerning mercury waste handling, environmental protection, and patient education. As such, amalgam reduction and elimination policies will depend first on the awareness, knowledge levels, and attitudes of dental professionals.

Previous studies showed variability among dentists on risk perception about amalgam with inconsistent results for environmental risks, occupational exposure risk, and patient safety.^3,8,10,24^ Furthermore, though awareness about the Minamata Convention has been described as moderate to low in many countries,^1,20^ data are limited and heterogeneous concerning updated policy decisions and their implications for day-to-day dental practice. To the best of our knowledge, no comprehensive study has evaluated Turkish dentists’ awareness and knowledge regarding the phase-down of dental amalgam, despite Türkiye being a party to the Minamata Convention. Accordingly, the present study aimed to assess the level of awareness and knowledge among dentists toward the Minamata Convention as well as toward gradual reduction of dental amalgam use. Results would be used to identify knowledge gaps and educational needs for environmentally sustainable dental practice.

This study tested the null hypothesis that dentists’ knowledge of and attitudes toward the Minamata Convention, the 2025 amalgam phase-down/phase-out regulatory decisions, and current dental amalgam use do not differ significantly across sociodemographic and professional subgroups.

## MATERIALS AND METHODS

This study was approved by the Scientific Research Ethics Committee of Çankırı Karatekin University (Decision No: 24, Date: 18/12/2025). The study was conducted in accordance with the ethical principles outlined in the Declaration of Helsinki and adhered to the Strengthening the Reporting of Observational Studies in Epidemiology (STROBE) guidelines. Informed consent to participate was obtained from all of the participants in the study.

The target population comprised all categories of dentists currently engaged in clinical or academic practice within Türkiye: general dental practitioners, specialists, postgraduate (PhD or residency) students, and university faculty. Participation was voluntary and contingent on the provision of informed consent. Respondents were excluded if their submissions were incomplete, identified as duplicates, or if they were not actively practising in Türkiye at the time of data collection. Sample size estimation was performed using the Raosoft calculator (Raosoft; Seattle, WA, USA) with the following parameters: 95% confidence level, 5% margin of error, and a reference population of 54,062 registered dentists in Türkiye as documented by the Turkish Dental Association in 2024.^25^ The calculation yielded a minimum requirement of 382 respondents. Anticipating a proportion of unusable or partially completed responses, recruitment continued until 408 valid questionnaires were obtained for the final analysis.

The questionnaire was developed on the basis of a thorough examination of the existing literature, including previously published studies on dental amalgam use, mercury awareness, and the Minamata Convention.^1,8,10,17^ The survey consisted of 26 questions organized into five sections: sociodemographic and professional characteristics (5 items), amalgam use (1 item), perceived health and environmental risks associated with amalgam (3 items), awareness of the Minamata Convention and the 2025 phase-down decision (2 items) along with the primary source of information on the topic (1 item), knowledge about dental amalgam and mercury-related regulations (8 items), and attitudes toward the amalgam phase-down process, environmental concerns, and continuing education needs (6 items). Content validity was established through five experts specialising in restorative dentistry and paediatric dentistry. Each item was rated on a four-point ordinal scale ranging from “not relevant” (1) to “highly relevant” (4) across three domains: relevance, clarity, and representativeness. At the item level, content validity indices (I-CVI) ranged from 0.80 to 1.00. The scale-level content validity index, calculated as the average across items (S-CVI/Ave), was 0.90 for the knowledge domain, 0.93 for the attitude domain, and 0.91 for the instrument as a whole values consistent with excellent content validity. Following expert review, the instrument was subsequently refined to enhance the precision and readability of individual items. All participants responded to the same instrument under standardized conditions, ensuring comparability of the data across groups. The data were based entirely on self-reported responses. The survey link was distributed between January and April 2026 through various social media platforms commonly used by dentists and via email to dentists registered with local dental chambers. The data collection process complied with the privacy regulations and usage terms of the hosting platform. Because the instrument did not solicit any personally identifiable information, respondent anonymity was preserved throughout the study.

Two primary outcome measures were defined. The first was the knowledge score, derived from eight factual items addressing the 2025 Minamata Convention decisions, and was assessed using an eight-item scale (score range: 0–8). The second was the attitude score, measured using a six-item Likert-type scale derived from items evaluating views on amalgam phase-down, environmental concerns, and the need for continuing education (score range: 1–5).

Secondary outcomes included the frequency of amalgam use, perceived health and environmental risks associated with amalgam, familiarity with the Minamata Convention, and awareness of the 2025 phase-down/phase-out regulatory updates.

The independent variables examined in this study were grouped into three categories. Sociodemographic variables included age and sex. Professional variables included professional title (general dentist, specialist dentist, academic staff, or postgraduate student), specialty field, institution type (private practice, public institution, or university), and years of professional experience. Practice- and awareness-related variables included frequency of amalgam use and self-reported awareness of the Minamata Convention and the 2025 phase-down decision.

Professional title and institution type were considered potential confounding factors in the interpretation of group comparisons, as both variables may independently influence knowledge and attitudes toward amalgam phase-down. The potential modifying effect of specialty field was also explored, given that different specialties may vary in their exposure to amalgam-related clinical practices and regulatory information. However, as no multivariable regression analysis was performed, these relationships were assessed through stratified bivariate comparisons rather than formal confounder adjustment.

### Statistical Analysis

All statistical analyses were performed using SPSS Statistics (IBM; Armonk, NY, USA). The significance level was set at p < 0.05.

Categorical variables were summarized as frequencies and percentages, and continuous variables were presented as mean ± standard deviation. A total of eight knowledge items were scored as correct (1) or incorrect (0), yielding a knowledge score ranging from 0 to 8. Participants were classified into three knowledge levels: low (0–2), moderate (3–5), and high (6–8). The attitude phase-down score was calculated as the mean of six Likert-type items (1 = strongly disagree to 5 = strongly agree) assessing attitudes toward dental amalgam phase-down, environmental awareness, and continuing education needs. Internal consistency was evaluated using Cronbach’s alpha.

Normality was assessed using the Shapiro–Wilk test and visual inspection of histograms and Q–Q plots. Homogeneity of variances was evaluated using Levene’s test. For two-group comparisons, the independent-samples t-test or Welch t-test was used depending on whether equality of variances was met. For multi-group comparisons, Welch’s ANOVA with Games–Howell post-hoc tests was used when the homogeneity assumption was violated. The association between knowledge and attitude scores was assessed using Pearson’s correlation coefficient with 95% confidence interval.

Cohen’s d was reported as the effect size for two-group comparisons and eta-squared (η²) for multi-group comparisons. No correction for multiple comparisons was applied given the exploratory nature of the analyses; accordingly, the possibility of inflated Type I error should be considered when interpreting the results.

## RESULTS

**Fig 1:**
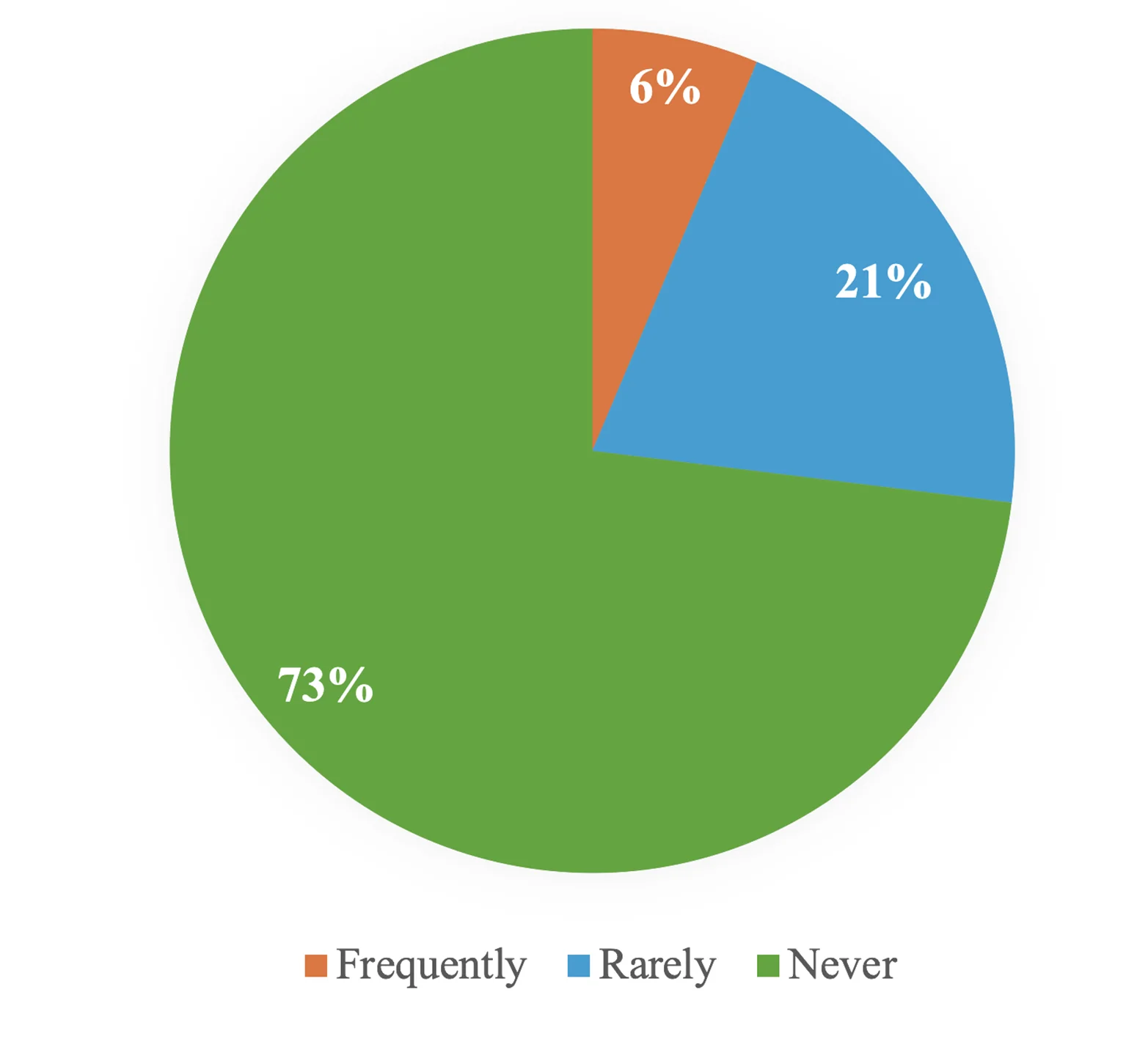
Frequency of dental amalgam use among dentists.

**Fig 2:**
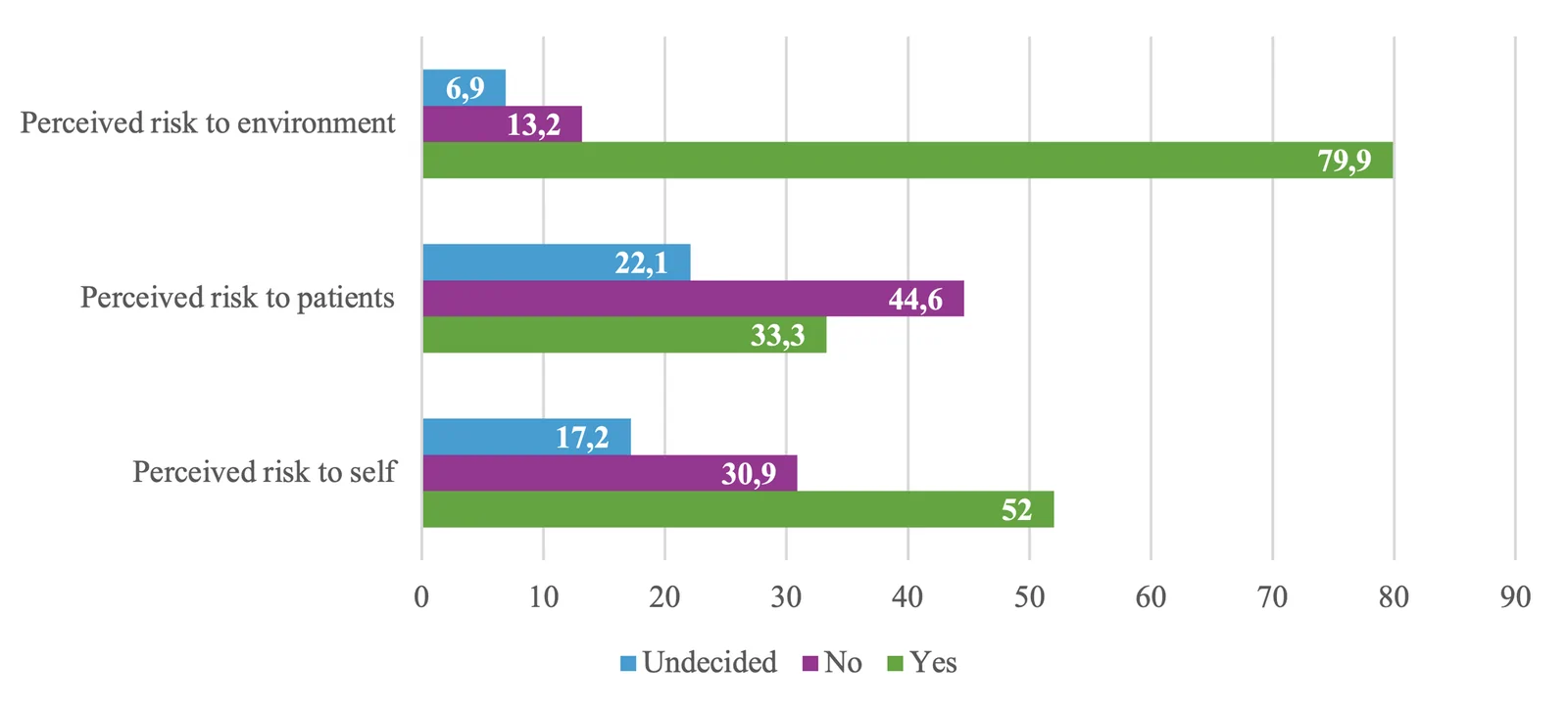
Comparison of perceived risks associated with dental amalgam for self, patients, and the environment.

**Fig 3:**
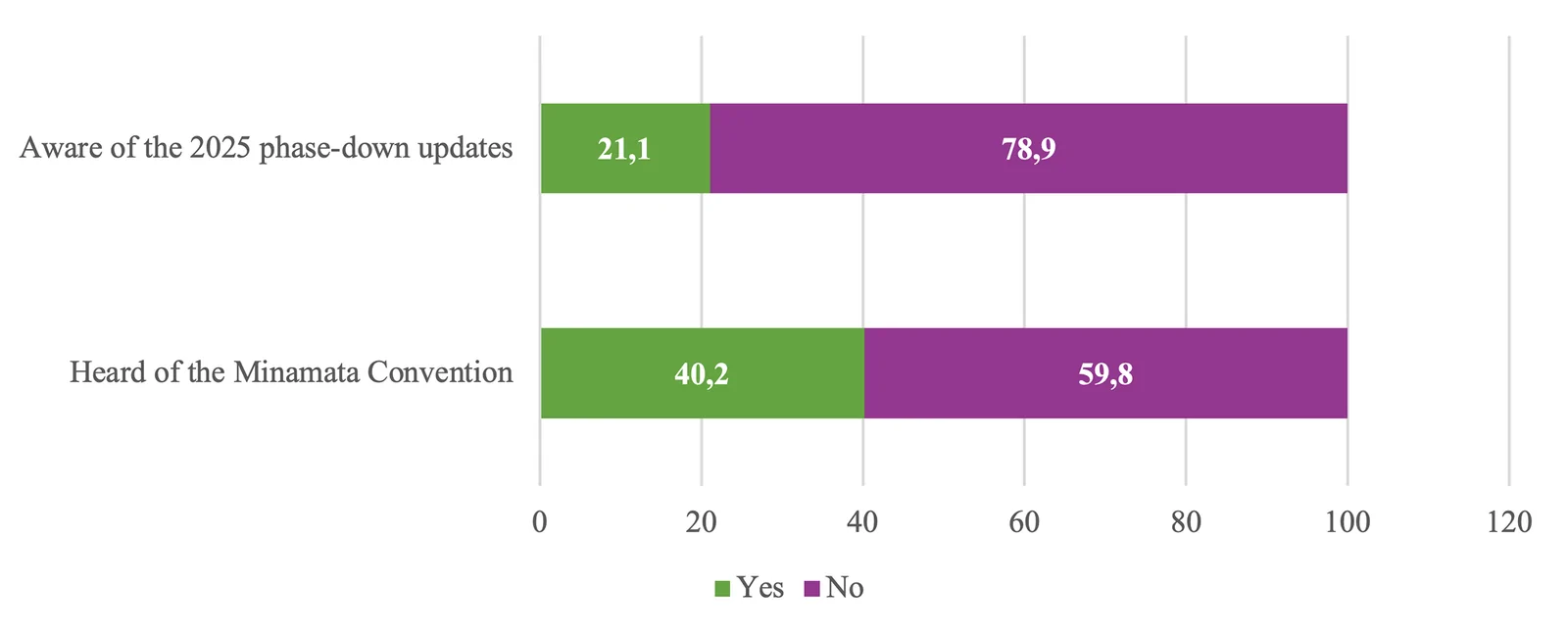
Awareness of the Minamata Convention and the 2025 phase-down updates among dentists.

**Fig 4:**
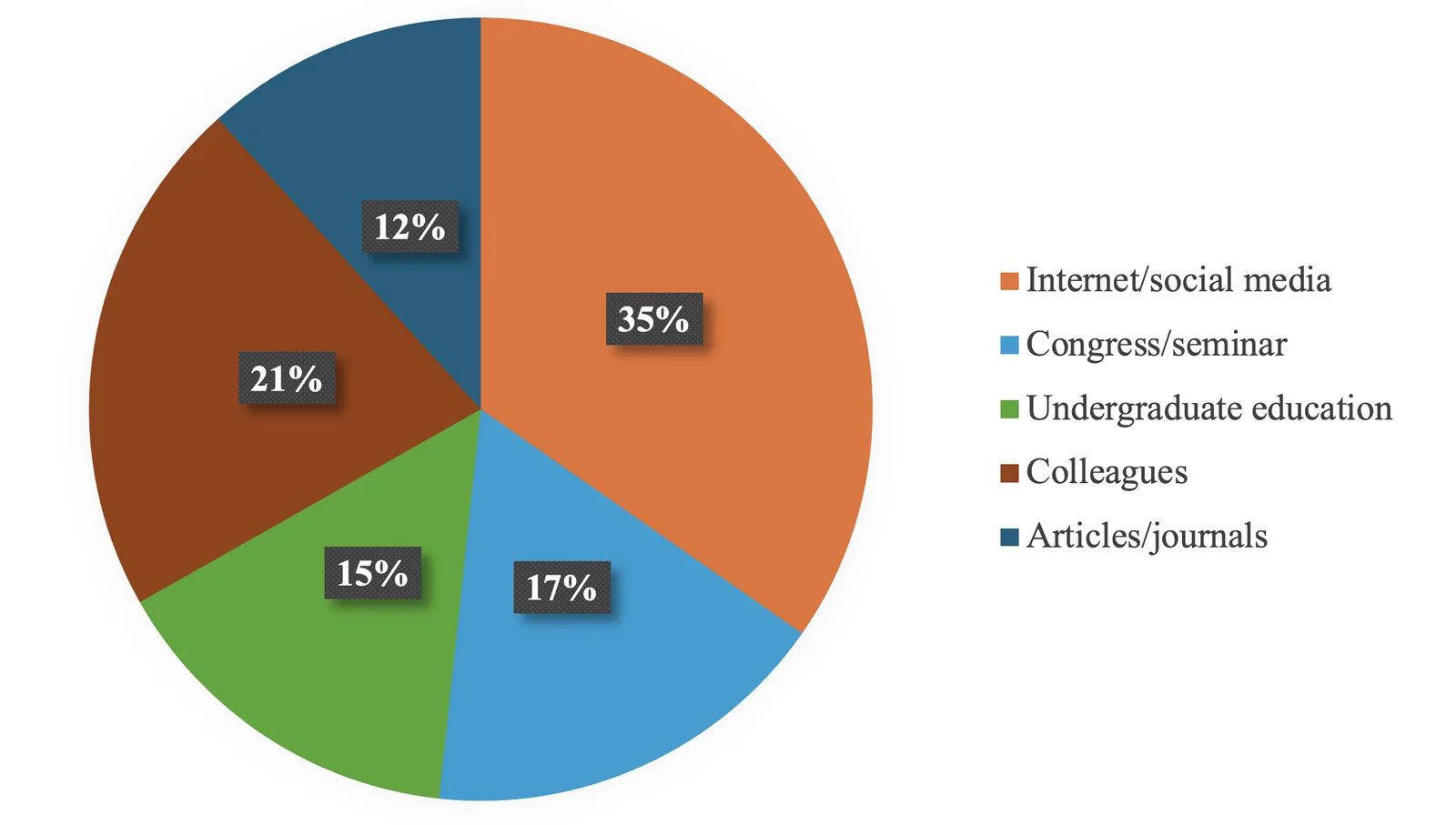
Sources of information regarding the Minamata Convention.

Table 1 presents the sociodemographic and professional profile of the study sample. The majority of participants (73.0%) reported never using dental amalgam, while 20.6% reported rare and 6.4% frequent use (Fig 1). Regarding perceived risks, amalgam was considered a risk to the environment by 79.9% of participants, to practitioners themselves by 52.0%, and to patients by 33.3% (Fig 2). A total of 40.2% of participants had heard of the Minamata Convention, whereas only 21.1% were aware of the 2025 amalgam phase-down updates under the Minamata Convention (Fig 3). The most frequently reported source of information was internet/social media (34.7%) (Fig 4).

**Table 1:** Demographic and professional characteristics of participants

Variable	Category	n	%
Age, years	Mean ± SD	39.14 ± 13.46	
	Range	23–76	
Sex	Female	241	59.1
	Male	167	40.9
Professional title	General dentist	214	52.5
	Specialist dentist	60	14.7
	Academic staff	74	18.1
	Postgraduate student	60	14.7
Institution	Private practice	205	50.2
	Public institution	71	17.4
	University	132	32.4
Professional experience	0–5 years	110	27.0
	6–10 years	110	27.0
	11–20 years	66	16.2
	>20 years	122	29.9


The distribution of correct and incorrect responses to the knowledge items and the mean scores of the attitude items are presented in Tables 2 and 3.

**Table 2:** Knowledge of the Minamata Convention among dentists

Knowledge item	Statement	Correct n (%)	Incorrect n (%)
K1	The primary aim of the Minamata Convention is to reduce exposure to mercury and mercury compounds.	230 (56.4%)	178 (43.6%)
K2	The 2025 Minamata Convention promotes a “phase-down” approach rather than a complete ban of dental amalgam.	238 (58.3%)	170 (41.7%)
K3	According to the 2025 decisions, dental amalgam is planned to be completely phased out by 2034.	174 (42.6%)	234 (57.4%)
K4	The 2025 update identifies children, pregnant, and breastfeeding individuals as priority groups for reducing amalgam use.	194 (47.5%)	214 (52.5%)
K5	Restricting the use of dental amalgam to encapsulated forms is defined as a key requirement for compliance.	134 (32.8%)	274 (67.2%)
K6	The promotion of alternative materials to dental amalgam is officially recommended in the 2025 revision.	194 (47.5%)	214 (52.5%)
K7	Türkiye is among the countries that are parties to the Minamata Convention.	92 (22.5%)	316 (77.5%)
K8	Dentists, health authorities, and educational institutions have responsibilities in implementing the Minamata Convention.	214 (52.5%)	194 (47.5%)


**Table 3:** Attitudes toward the phase-down of dental amalgam among dentists

Attitude item	Statement	Mean ± SD
A1	Modern alternative materials can provide adequate substitutes for dental amalgam in most cases.	4.03 ± 1.11
A2	The phase-down of dental amalgam under the Minamata Convention is necessary for environmental protection.	4.12 ± 1.14
A3	The gradual reduction of dental amalgam in line with the 2025 Minamata decisions is necessary.	4.00 ± 1.27
A4	Dentists and dental students should have sufficient knowledge about the Minamata Convention.	4.28 ± 1.12
A5	The Turkish Dental Association should provide more information to dentists about the Minamata Convention and the environmental impact of amalgam.	4.38 ± 1.02
A6	More educational programs should be organized for dentists regarding the Minamata Convention and alternative materials.	4.31 ± 1.02
Attitude items were scored on a 5-point Likert scale, where 1 = strongly disagree and 5 = strongly agree.		

The mean knowledge score was 3.60 ± 3.00 out of 8, with scores ranging from 0 to 8. The distribution of knowledge scores was highly dispersed, with a coefficient of variation of 83.3%. Overall, 41.7% of participants were classified as having low knowledge, 21.6% as having moderate knowledge, and 36.8% as having high knowledge, suggesting a polarized distribution across knowledge levels. Among individual knowledge items, the highest correct response rate was observed for K2 (58.3%), whereas the lowest correct response rate was observed for K7 (22.5%).

The six-item attitude phase-down scale, consisting of A1, A2, A3, A4, A5, and A6, demonstrated high internal consistency (Cronbach’s α = 0.889). The mean attitude phase-down score was 4.19 ± 0.89 on a 5-point scale.

A weak positive correlation was observed between knowledge score and attitude phase-down score, r = 0.242, 95% CI: 0.15–0.33, p < 0.001. Higher knowledge scores were associated with higher attitude phase-down scores.

When knowledge was analyzed categorically, attitude phase-down scores differed significantly across knowledge levels. Because Levene’s test indicated heterogeneous variances, Welch’s ANOVA with Games–Howell post hoc comparisons was used. Welch’s ANOVA showed a statistically significant difference among knowledge-level groups, Welch’s F(2, 245.911) = 11.941, p < 0.001. Games–Howell post hoc comparisons indicated that participants with low knowledge had statistically significantly lower attitude scores than those with moderate knowledge (3.93 ± 1.00 vs. 4.32 ± 0.69, p = 0.001) and high knowledge (3.93 ± 1.00 vs. 4.40 ± 0.80, p < 0.001), whereas no significant difference was observed between moderate- and high-knowledge groups (p = 0.678).

Group comparisons are presented in Table 4. Knowledge scores did not differ significantly according to sex, t(406) = 1.298, p = 0.195, Cohen’s d = 0.13. However, attitude phase-down scores were statistically significantly higher among female participants than male participants, t(249.692) = 5.595, p < 0.001, Cohen’s d = 0.61.

**Table 4:** Comparison of knowledge and attitude scores according to participant characteristics

Variable	Group	Knowledge score, Mean ± SD	Effect size	p-value	Attitude score, Mean ± SD	Effect size	p-value
Sex	Female	3.76 ± 2.98	d = 0.13	0.195	4.40 ± 0.66	d = 0.61	<0.001
	Male	3.37 ± 3.04			3.88 ± 1.08		
Professional title	General dentist	3.03 ± 3.00	η² = 0.055	<0.001	4.07 ± 0.92	η² = 0.025	0.010
	Specialist dentist	3.57 ± 3.14			4.33 ± 0.89		
	Academic staff	4.81 ± 2.68			4.41 ± 0.73		
	Postgraduate student	4.20 ± 2.74			4.21 ± 0.92		
Institution	Private practice	2.84 ± 2.95	η² = 0.066	<0.001	4.18 ± 0.93	η² = 0.010	0.113
	Public institution	4.13 ± 3.13			4.03 ± 0.88		
	University	4.50 ± 2.71			4.29 ± 0.83		
Professional experience	0–5 years	2.84 ± 2.83	η² = 0.028	0.005	4.15 ± 1.00	η² = 0.032	0.003
	6–10 years	4.16 ± 2.66			4.37 ± 0.60		
	11–20 years	3.70 ± 3.14			3.88 ± 1.01		
	>20 years	3.74 ± 3.25			4.23 ± 0.92		
Amalgam use frequency	Frequently	3.77 ± 2.60	η² = 0.001	0.889	3.47 ± 0.83	η² = 0.069	<0.001
	Rarely	3.69 ± 2.68			3.96 ± 0.83		
	Never	3.56 ± 3.13			4.32 ± 0.88		
Heard of the Minamata Convention	Yes	5.49 ± 2.25	d = 1.22	<0.001	4.36 ± 0.81	d = 0.32	0.001
	No	2.34 ± 2.77			4.08 ± 0.93		
Aware of the 2025 amalgam phase-down updates	Yes	6.21 ± 1.89	d = 1.23	<0.001	4.37 ± 0.83	d = 0.26	0.032
	No	2.91 ± 2.86			4.14 ± 0.90		
Cohen’s d was reported for two-group comparisons. η² was reported for multi-group comparisons and was calculated from the ANOVA sum of squares. Welch’s ANOVA with Games–Howell post-hoc comparisons was used for multi-group comparisons when Levene’s test indicated heterogeneous variances. For two-group comparisons, the Welch t-test result was interpreted when equality of variances was violated.							

Knowledge scores differed significantly according to professional title, Welch’s F(3, 145.454) = 8.431, p < 0.001, η² = 0.055. Games–Howell post-hoc comparisons showed that academic staff (4.81 ± 2.68) and postgraduate students (4.20 ± 2.74) had statistically significantly higher knowledge scores than general dentists (3.03 ± 3.00) (p < 0.001 and p = 0.025, respectively). Attitude phase-down scores also differed statistically significantly according to professional title, Welch’s F(3, 146.215) = 3.892, p = 0.010, η² = 0.025. Post hoc comparisons indicated that academic staff had significantly higher attitude scores than general dentists (4.41 ± 0.73 vs. 4.07 ± 0.92, p = 0.008), whereas the remaining pairwise comparisons were not statistically significant.

Institution type was significantly associated with knowledge scores, Welch’s F(2, 181.263) = 14.896, p < 0.001, η² = 0.066. Games–Howell post-hoc comparisons showed that participants working in private practice had significantly lower knowledge scores (2.84 ± 2.95) than those working in public institutions (4.13 ± 3.13, p = 0.009) and universities (4.50 ± 2.71, p < 0.001). No significant difference was observed between public institutions and universities (p = 0.674). Attitude phase-down scores did not differ significantly according to institution type, Welch’s F(2, 187.809) = 2.209, p = 0.113, η² = 0.010.

Professional experience was statistically significantly associated with both knowledge and attitude scores. Knowledge scores differed significantly according to professional experience, Welch’s F(3, 200.929) = 4.373, p = 0.005, η² = 0.028. Games–Howell post hoc comparisons showed that participants with 6–10 years of professional experience had significantly higher knowledge scores than those with 0–5 years of experience (4.16 ± 2.66 vs 2.84 ± 2.83, p = 0.002). No other pairwise comparisons were statistically significant. Attitude phase-down scores also differed significantly according to professional experience, Welch’s F(3, 193.058) = 4.814, p = 0.003, η² = 0.032. Games–Howell post-hoc comparisons indicated that participants with 6–10 years of experience had statistically significantly higher attitude scores than those with 11–20 years of experience (4.37 ± 0.60 vs. 3.88 ± 1.01, p = 0.003). The remaining pairwise comparisons were not statistically significant.

Knowledge scores did not differ significantly according to amalgam-use frequency, Welch’s F(2, 65.555) = 0.118, p = 0.889, η² = 0.001. In contrast, attitude phase-down scores differed significantly among amalgam-use groups, Welch’s F(2, 63.074) = 15.655, p < 0.001, η² = 0.069. Participants who never used amalgam had the highest attitude scores (4.32 ± 0.88), followed by those who used it rarely (3.96 ± 0.83) and frequently (3.47 ± 0.83). Post hoc comparisons showed statistically significant differences between frequent and rare users (p = 0.034), frequent users and non-users (p < 0.001), and rare users and non-users (p = 0.002).

Participants who had previously heard of the Minamata Convention had statistically significantly higher knowledge scores than those who had not heard of it, t(391.885) = 12.616, p < 0.001, Cohen’s d = 1.22. Similarly, their attitude phase-down scores were statistically significantly higher, t(380.011) = 3.210, p = 0.001, Cohen’s d = 0.32.

Participants who were aware of the 2025 amalgam phase-down/phase-out process had statistically significantly higher knowledge scores than those who were not aware, t(201.472) = 12.781, p < 0.001, Cohen’s d = 1.23. Similarly, attitude phase-down scores were statistically significantly higher among participants who were aware of the 2025 phase-down/phase-out process, t(406) = 2.149, p = 0.032, Cohen’s d = 0.26.

## DISCUSSION

The present study evaluated Turkish dentists’ awareness, knowledge, and attitudes regarding the Minamata Convention on Mercury and the regulatory decisions adopted at the Sixth Meeting of the Conference of the Parties (COP-6) in Geneva in November 2025. The findings reveal that awareness and factual knowledge of the Convention remain limited among Turkish dentists, despite Türkiye’s ratification of the treaty in 2022. In contrast, attitudes toward the amalgam phase-down were notably favorable across the sample. The null hypothesis that knowledge and attitude scores do not differ across sociodemographic and professional subgroups was rejected, as significant differences were observed according to professional title, institution type, sex, amalgam use frequency, professional experience, and prior awareness of the Convention.

The COP-6 decisions represent a landmark regulatory shift in the global management of dental amalgam.^28^ Through Decision MC-6/3, dental amalgam was elevated from a phase-down to a phase-out product, with a legally binding 2034 phase-out deadline, while preserving clinical discretion when its use is considered necessary based on patient needs. Against this regulatory background, one of the most striking findings of the present study was the low level of awareness regarding the Minamata Convention and its COP-6 amendments among Turkish dentists. Only 40.2% of the participants had heard of the Convention, and merely 21.1% were aware of the 2025 phase-down decisions. These figures are particularly concerning given that Türkiye officially ratified the Convention in October 2022 and is now legally bound by the obligations set forth in the amended Annex A, including the 2034 phase-out timeline and the requirement to submit national action plans. The finding that only 22.5% of participants correctly identified Türkiye as a party to the Convention (K7) the lowest correct response rate among all knowledge items further underscores this awareness gap. The item with the second-lowest correct response rate was The second-lowest correct response rate was observed for item K5 (32.8%), indicating limited awareness regarding the restriction of non-encapsulated dental amalgam use. These results are broadly consistent with earlier studies conducted in other countries. These results are broadly consistent with, although notably more favorable than, findings from comparable studies conducted in other countries. Convention (K7) the lowest correct response rate among all knowledge items further underscores this awareness gap. The second-lowest correct response rate was observed for K5 (32.8%), reflecting limited awareness of the encapsulated-forms requirement. These results are broadly consistent with, although notably more favorable than, findings from comparable studies conducted in other countries. In Jordan, AL-Rabab’ah et al^1^ surveyed 196 randomly selected dentists and reported that only 13.8% were aware of the Minamata Convention a figure substantially lower than the 40.2% observed in the present study. Furthermore, only 28.1% of the Jordanian sample favored discontinuing amalgam use due to its health and environmental hazards, and statistically significant gaps were identified in undergraduate training for posterior composite placement, including inadequate training in rubber dam use and sectional matrix techniques. In Nigeria, Makanjuola et al^20^ conducted a nationwide survey of 845 dental students and dentists across all geopolitical zones and found that 87.7% had poor knowledge of the Minamata Convention, while 72.0% lacked training in the use of alternatives to amalgam. Moreover, amalgam continued to be commonly used by 39.1% of dental students and 31.3% of dentists in Nigeria, and only 4.7% of participants reported following good amalgam phase-down practices. Awareness and phase-down practices were significantly higher among dentists than among dental students. These cross-national comparisons suggest that while Turkish dentists demonstrate relatively higher awareness than their Jordanian and Nigerian counterparts, substantial knowledge gaps persist across all settings and highlight the global need for targeted educational interventions to support the transition mandated by the COP-6 amendments.

The overall mean knowledge score (3.60 ± 3.00 out of 8) indicates a moderate-to-low level of knowledge across the sample. However, the high coefficient of variation (83.3%) and the distribution of participants across knowledge categories 41.7% low, 21.6% moderate, and 36.8% high reveal a markedly polarized rather than normally distributed knowledge profile. This polarization suggests that a distinct subgroup of dentists, primarily those in academic settings, possesses substantial knowledge, while the majority, particularly general practitioners in private practice, have limited understanding of the Convention and its implications. The finding that private practice dentists had the lowest mean knowledge score (2.84 ± 2.95) compared with those in public institutions (4.13 ± 3.13) and universities (4.50 ± 2.71) supports this interpretation. This is particularly relevant given that the COP-6 amendments to Part II of Annex A specifically call for encouraging representative professional organizations and dental schools to educate and train dental professionals and students on the use of mercury-free dental restoration alternatives. The observation that internet and social media were the most commonly cited information source (34.7%), rather than formal professional channels such as dental chambers or structured continuing education programs, suggests that these formal educational mechanisms have not yet been effectively mobilized. These findings are also consistent with, and extend, the results of previous studies conducted among Turkish dentists. Erçin et al^10^ surveyed 1211 dentists and 320 patients in Türkiye and reported that 63.1% of dentists did not use amalgam at all, while 24.0% used it routinely and 9.5% rarely — a pattern broadly comparable to the present study, in which 73.0% reported never using amalgam. Notably, the primary reasons for avoiding amalgam cited by Erçin et al^10^ were its unaesthetic properties and patient preference rather than environmental or regulatory concerns, and dentists in that study generally disagreed on banning dental amalgam despite its low utilization. This suggests that the decline in amalgam use in Türkiye has been driven predominantly by esthetic and patient-related factors rather than by awareness of the Minamata Convention an interpretation further supported by our finding that knowledge scores did not differ significantly according to amalgam use frequency. More recently, Çöl et al^8^ surveyed 269 dentists in Türkiye and found that although 97.2% had received undergraduate training on amalgam, only 23.6% were currently using it in practice. Importantly, 39.6% of participants reported that their knowledge of safe amalgam removal protocols was insufficient or uncertain, and only 45.6% expressed concern about the environmental impact of mercury. These figures are lower than the 79.9% environmental risk perception observed in the present study, a discrepancy that may reflect differences in question framing, sample composition, or a growing awareness trend in the intervening period.

The analysis of professional subgroups revealed several noteworthy patterns. Academic staff and postgraduate students demonstrated significantly higher knowledge scores than general dentists, a finding attributable to their closer engagement with the scientific literature, participation in research activities, and institutional emphasis on evidence-based practice. Regarding professional experience, participants with 6–10 years of experience had statistically significantly higher knowledge and attitude scores than their less experienced counterparts (0–5 years). This finding may reflect a cohort that has accumulated sufficient clinical experience while remaining temporally proximate to their formal education, and therefore more likely to be engaged with current professional developments. The relatively lower knowledge scores among the 0–5 year group raise questions about the extent to which the Minamata Convention and mercury-related environmental regulations have been integrated into undergraduate dental curricula in Türkiye. Given that the COP-6 amendments explicitly encourage dental schools to educate students on mercury-free alternatives and best management practices, curricular reform appears to be an urgent priority.

A statistically significant sex-based difference was observed in attitude scores, with female participants scoring significantly higher than males (Cohen’s d = 0.61, a medium effect size), whereas no statistically significant difference was found in knowledge scores. This pattern of similar knowledge but more favorable attitudes among female practitioners is consistent with broader health behavior literature indicating that women tend to demonstrate greater sensitivity toward environmental and preventive health concerns.^9,13^ This finding also has relevance in the context of the COP-6 framework, which specifically addresses the health concerns of women and children through Decision MC-6/16, and identifies pregnant and breastfeeding women as a priority population for amalgam use restrictions.

The weak but statistically significant positive correlation between knowledge and attitude scores (r = 0.242, p < 0.001) merits careful interpretation. While higher knowledge was associated with more favorable attitudes, the modest magnitude of this relationship indicates that positive attitudes toward amalgam reduction can exist largely independently of factual knowledge about the Convention. Indeed, the overall mean attitude score was high (4.19 ± 0.89 out of 5.00) even though a substantial proportion of participants had limited knowledge. This apparent knowledge–attitude dissociation may reflect a general professional inclination toward environmentally responsible practice that is not necessarily grounded in specific regulatory awareness. Nevertheless, when knowledge was analysed categorically, participants in the low-knowledge group had significantly lower attitude scores than those in the moderate- and high-knowledge groups, suggesting that a minimum threshold of knowledge may be necessary to sustain favorable attitudes. This finding underscores the potential value of targeted educational interventions.

The analysis of amalgam use patterns revealed that the majority of participants (73.0%) never used dental amalgam in their current practice, while 79.9% perceived it as an environmental risk. These findings suggest that the practical shift away from amalgam in Türkiye has largely already occurred, likely driven by the widespread adoption of resin-based composites and other tooth-colored alternatives rather than by conscious regulatory compliance. This observation is noteworthy in relation to Decision MC-6/10, which recognized dental amalgam as the largest remaining use of mercury in mercury-added products globally a characterization that may apply more to certain regions than to countries like Türkiye where amalgam use has already substantially declined. However, the statistically significant association between amalgam use frequency and attitude scores with non-users demonstrating the most favorable attitudes (4.32 ± 0.88) and frequent users the least favorable (3.47 ± 0.83) indicates that continued amalgam use may be linked to more resistant attitudes toward phase-down. This finding has practical implications for the design of transition programs, as the COP-6 amendments require parties to take measures to not allow, or statistically significantly phase down, dental amalgam unless its use is considered necessary by the dental practitioner based on the needs of the patient. Practitioners who still rely on amalgam may require more intensive engagement and support to adopt alternative materials while maintaining clinical standards. Notably, knowledge scores did not differ statistically significantly according to amalgam use frequency, indicating that the decision to use or avoid amalgam is influenced more by attitudinal and practical factors than by regulatory knowledge.

The finding that participants who were aware of both the Minamata Convention (Cohen’s d = 1.22) and the 2025 phase-down updates (Cohen’s d = 1.23) had substantially higher knowledge scores, with large effect sizes, is expected given the conceptual overlap between awareness and factual knowledge. More importantly, awareness was also associated with statistically significantly higher attitude scores, though with smaller effect sizes (d = 0.32 and d = 0.26, respectively). This suggests that even basic exposure to information about the Convention may contribute to more favorable attitudes, reinforcing the rationale for broad dissemination campaigns as the 2034 phase-out deadline approaches.

The individual knowledge items provide further insight into specific areas of deficiency that are directly relevant to the COP-6 framework. While 58.3% correctly identified the phase-down approach (K2) and 56.4% recognized the Convention’s primary aim (K1), only 42.6% were aware of the 2034 complete phase-out deadline (K3), 47.5% knew about priority populations for reducing amalgam use (K4), and 32.8% were aware of the amalgam separator requirement (K5). These knowledge gaps are concerning because each of these items corresponds to specific binding obligations established or reinforced by Decision MC-6/3. In particular, the low awareness of the 2034 phase-out date now a binding timeline for all parties for which the amendment enters into force suggests that the regulatory shift from phase-down to phase-out has not been effectively communicated to the dental profession in Türkiye. Similarly, the limited awareness of priority populations (children under 15, pregnant and breastfeeding women) is significant, given that the COP-6 amendments explicitly mandate parties to exclude or recommend against amalgam use in these groups, except when considered necessary by the dental practitioner based on the needs of the patient.

The attitude items revealed consistently high scores across all domains, with the highest agreement observed for A5 (4.38 ± 1.02), which endorsed the need for the Turkish Dental Association to provide more information about the Minamata Convention, and A6 (4.31 ± 1.02), which supported the development of educational programs. These findings reflect a clear demand from the profession itself for institutional guidance and educational support a demand that aligns directly with the COP-6 amendments to Part II of Annex A, which call for encouraging representative professional organizations and dental schools to educate and train dental professionals on mercury-free alternatives and best management practices.

Taken together, these findings suggest a need for greater dissemination of information regarding the Minamata Convention and the COP-6 amendments among Turkish dentists. This need is supported by the high agreement with items A5 and A6, which emphasized the role of professional organizations and educational programs. Lower knowledge scores among general practitioners in private practice indicate that this group may particularly benefit from future awareness and educational initiatives. In addition, the prominence of internet and social media as information sources highlights the potential value of digital communication channels.

This study has several limitations that should be considered when interpreting the results. First, the cross-sectional design precludes causal inferences regarding the relationships between knowledge, attitudes, and sociodemographic variables. Second, the convenience sampling strategy, relying on social media distribution and email invitations through dental chambers, may introduce selection bias favoring digitally active or professionally engaged dentists, potentially overestimating awareness and knowledge levels in the broader population. Third, all data were self-reported, and responses may be subject to social desirability bias, particularly for attitude items. Fourth, data collection commenced shortly after the COP-6 decisions in November 2025, and awareness levels may change as the regulatory implications become more widely communicated and as Türkiye develops its national action plan for amalgam phase-out. Longitudinal studies would be valuable to track changes in knowledge and attitudes as the 2034 phase-out deadline approaches.

## CONCLUSION

In this cross-sectional survey of 408 dentists in Türkiye, awareness of the Minamata Convention and the 2025 COP-6 amendments was limited, while knowledge gaps were more pronounced among general practitioners in private practice and early-career dentists. In contrast, attitudes toward amalgam phase-down were broadly favorable across the sample, with female participants demonstrating statistically significantly more positive attitudes. Knowledge and attitude scores were weakly but statistically significantly correlated. Participants most frequently identified internet and social media as their primary information source and expressed high agreement with the need for further guidance from the Turkish Dental Association and structured educational programs on the Convention and mercury-free alternatives.
